# Supplementary material for: Foetal gluten immunogenic peptides during pregnancy: a new determinant on the coeliac exposome
Source: BMC Med. 2024 Jul 18;22:295. doi: 10.1186/s12916-024-03495-9 (PMC11256569; doi:10.1186/s12916-024-03495-9)
Supplement: Supplementary file 1 — Additional file 1: Table S1 Clinical characteristics of pregnant women and new-born undergoing amniocentesis test (n = 33). CS, caesarean section; IVF, in vitro fecundation; VD, vaginal delivery; w, week; y, year [file 12916_2024_3495_MOESM1_ESM.pdf]

<table>
<tr><th colspan="3">Fetal samples</th><th>Volunteer</th><th colspan="4">Pregnancy</th><th colspan="4">Delivery</th></tr>
<tr><th>Status</th><th>Code</th><th>Gestational age, w</th><th>Age, y</th><th colspan="2">Single/twin</th><th>IVF/ Spontaneous conception</th><th>Interruption of pregnancy</th><th>Mode</th><th>New-born status</th><th>Gestational age, w</th><th>Premature rupture of membranes</th></tr>
<tr><td rowspan="14">Healthy</td><td>V5</td><td>21</td><td>28</td><td colspan="2" rowspan="11">Single</td><td rowspan="9">Spontaneous</td><td rowspan="14">No</td><td rowspan="11">VD</td><td rowspan="14">Healthy</td><td>40</td><td rowspan="7">No</td></tr>
<tr><td>V6</td><td>19</td><td>18</td><td>39</td></tr>
<tr><td>V14</td><td>16</td><td>40</td><td>40</td></tr>
<tr><td>V16</td><td>21</td><td>20</td><td>40</td></tr>
<tr><td>V26</td><td>16</td><td>37</td><td>39</td></tr>
<tr><td>W5</td><td>17</td><td>40</td><td>40</td></tr>
<tr><td>W7</td><td>20</td><td>36</td><td>39</td></tr>
<tr><td>V18</td><td>19</td><td>38</td><td>37</td><td rowspan="2">Yes</td></tr>
<tr><td>V29</td><td>16</td><td>39</td><td>40</td></tr>
<tr><td>V10</td><td>17</td><td>32</td><td rowspan="2">IVF</td><td>39</td><td rowspan="5">No</td></tr>
<tr><td>V24</td><td>16</td><td>43</td><td>39</td></tr>
<tr><td>V15</td><td>19</td><td>39</td><td rowspan="3">Twin</td><td rowspan="2">Monoamniotic</td><td rowspan="3">Spontaneous</td><td rowspan="3">CS
Twin pregnancy</td><td>38</td></tr>
<tr><td>W3</td><td>28</td><td>28</td><td>38</td></tr>
<tr><td>W3</td><td>18</td><td>36</td><td>Diamniotic</td><td>34</td></tr>
<tr><td rowspan="13">Malformations</td><td>V20</td><td>20</td><td>31</td><td colspan="2" rowspan="12">Single</td><td rowspan="11">Spontaneous</td><td rowspan="7">No</td><td rowspan="2">VD</td><td rowspan="3">Malformation</td><td>40</td><td rowspan="6">No</td></tr>
<tr><td>V28</td><td>20</td><td>32</td><td>40</td></tr>
<tr><td>V12</td><td>21</td><td>30</td><td rowspan="5">CS</td><td>40</td></tr>
<tr><td>V21</td><td>22</td><td>38</td><td rowspan="2">Growth retardation</td><td>32</td></tr>
<tr><td>V34</td><td>18</td><td>32</td><td>29</td></tr>
<tr><td>V27</td><td>20</td><td>28</td><td rowspan="2">Fetal heart disease</td><td>37</td></tr>
<tr><td>W4</td><td>21</td><td>36</td><td>40</td><td>Yes</td></tr>
<tr><td>V2</td><td>19</td><td>37</td><td rowspan="5">Yes</td><td colspan="4" rowspan="5"></td></tr>
<tr><td>W1</td><td>22</td><td>20</td></tr>
<tr><td>W2</td><td>21</td><td>31</td></tr>
<tr><td>W6</td><td>22</td><td>39</td></tr>
<tr><td>V9</td><td>21</td><td>33</td><td>IVF</td></tr>
<tr><td>V32</td><td>30</td><td>30</td><td>Twin</td><td>Monoamniotic</td><td>Spontaneous</td><td>No</td><td>CS
Twin pregnancy</td><td>Malformation</td><td>36</td><td>No</td></tr>
<tr><td rowspan="6">Chromosomopathies</td><td>V1</td><td>24</td><td>28</td><td colspan="2" rowspan="5">Single</td><td rowspan="5">Spontaneous</td><td rowspan="4">Yes</td><td rowspan="4"></td><td rowspan="6">Down´s syndrome</td><td rowspan="4"></td><td rowspan="4"></td></tr>
<tr><td>V4</td><td>19</td><td>39</td></tr>
<tr><td>V7</td><td>22</td><td>37</td></tr>
<tr><td>V13</td><td>16</td><td>44</td></tr>
<tr><td>V19</td><td>19</td><td>32</td><td>No</td><td>VD</td><td>38</td><td>Yes</td></tr>
<tr><td>V3</td><td>16</td><td>39</td><td>Twin</td><td>Diamniotic</td><td>IVF</td><td>Yes</td><td></td><td></td><td></td></tr>
</table>
